# CodingDiv: analyzing SNP-level microdiversity to discriminate between coding and noncoding regions in viral genomes

**DOI:** 10.1093/bioinformatics/btad408

**Published:** 2023-07-14

**Authors:** Eric Olo Ndela, François Enault

**Affiliations:** Université Clermont Auvergne, CNRS, LMGE, F-63000 Clermont-Ferrand, France; Université Clermont Auvergne, CNRS, LMGE, F-63000 Clermont-Ferrand, France

## Abstract

**Summary:**

Viral genes, that are frequently small genes and/or with large overlaps, are still difficult to predict accurately. To help predict all genes in viral genomes, we provide CodingDiv that detects SNP-level microdiversity of all potential coding regions, using metagenomic reads and/or similar sequences from external databases. Protein coding regions can then be identified as the ones containing more synonymous SNPs than unfavorable nonsynonymous substitutions SNPs.

**Availability and implementation:**

CodingDiv is released under the GPL license. Source code is available at https://github.com/ericolo/codingDiv. The software can be installed and used through a docker container.

## 1 Introduction

Viral genomes often encode small, overlapping, and even overprinting genes (Pavesi *et al.* 2013). Gene prediction softwares usually overlook these genes (Duval and Cossart 2017) as (i) short genes (<150 nt) are difficult to discriminate from random noncoding Open Reading Frames (ORFs) and (ii) large overlaps are penalized or even forbid (Hyatt *et al.* 2010). Predicting all the genes encoded in a genome is therefore currently not possible using only the genome itself. Additional sequences, similar to the genome of interest, can provide useful information. Once detected and aligned to the genome, such sequences provide information on codon substitutions and gap patterns that help identify coding regions (Claverie and Santini 2021). Implemented in softwares like RNAcode (Washietl *et al.* 2011) or PhyloCSF (Lin *et al.* 2011, Pockrandt *et al.* 2022), this strategy recently managed to identify >40 000 groups of small viral proteins from thousands of metagenomes (Fremin *et al.* 2022). Yet, finding several sequences closely related to a contig or genome is an uncertain task due to the great genomic diversity of viruses (Roux *et al.* 2015). Furthermore, it involves comparing the sequences of interest to the largest possible external databases. Here, we describe CodingDiv, a tool that discriminates coding from noncoding regions using SNP-level microdiversity, naturally occurring in viral populations from the environment (Gregory *et al.* 2019).

This microdiversity can be directly extracted from metagenomic reads from which the genome of interest was assembled. CodingDiv can also incorporate additional contigs of closely related species found in external databases.

## 2 Materials and methods

CodingDiv first predicts ORFs using getorf from the EMBOSS suite (Rice *et al.* 2000) and proteins using Prodigal (Hyatt *et al.* 2010) and PHANOTATE (McNair *et al.* 2019). Then, metagenomic reads, or contigs of related genomes, are mapped onto the reference genome using BWA mem (Li and Durbin 2009). SNP calling is done with the bcftools mpileup and call commands successively (Li *et al.* 2009). Classically, selective constraints of each ORF are quantified by the ratio of nonsynonymous to synonymous SNPs (pN/pS). Yet, nonsynonymous mutations with positive substitution scores in the BLOSUM62 matrix can be abundant in coding regions and also occur frequently in random noncoding regions. A ratio was thus computed considering only negative score mutations and synonymous ones (pNeg/pS, see Olo Ndela *et al.* 2023). Results are provided as two files and are used to generate a plot in SVG format.

### 2.1 Implementation and availability

CodingDiv is a shell script that runs pre-cited softwares and homemade R and Python scripts, using specific libraries such as DNA features viewer (Zulkower and Rosser 2020). CodingDiv can be installed via a docker container with all needed softwares and packages.

## 3 Results

**Figure 1.**
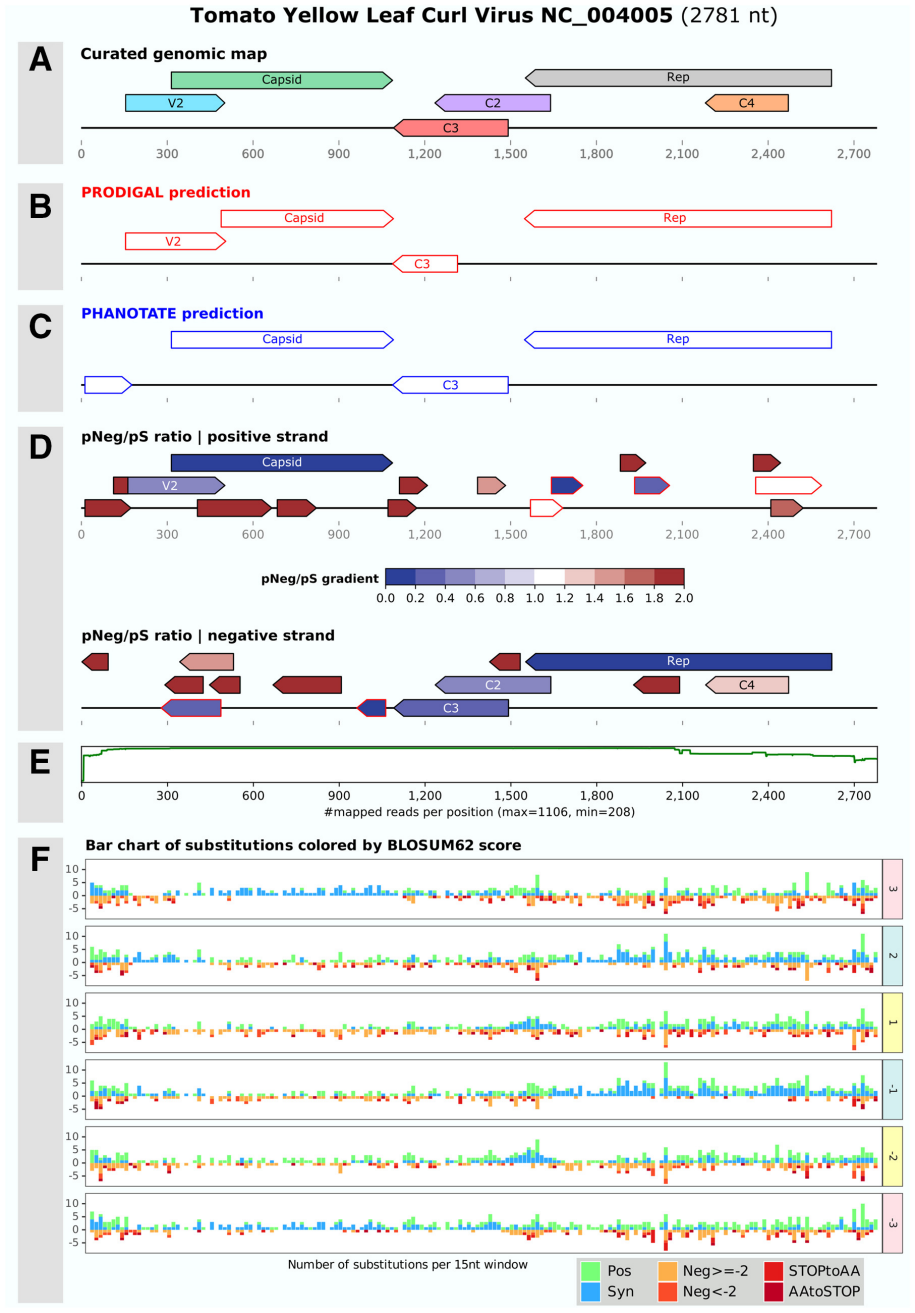
The image, in SVG format, was generated by CodingDiv with TYLCV virus as reference (NC_004005). A total of 1106 similar sequences collected from the nr database (>90% nucleotide identity) were used to identify SNPs (2 reads minimum, representing 1% of sequencing depth). The curated genomic map of this cultivated virus, as described in the literature, was added at the top of the image (A). Protein-coding genes predicted by Prodigal (B) and PHANOTATE (C), ORF prediction (>90 nt) colored according to their pNeg/pS ratio, on the two strands of the genome (D), a plot of the mapping coverage (E) and six bar charts (one per strand) counting substitutions from each SNP category per 15 nt window are shown (F). SNP were categorized as Synonymous or according to the induced substitution scores in the BLOSUM62 matrix.

To illustrate its relevance, CodingDiv was applied to the genome of a cultivated ssDNA eukaryotic virus, the Tomato Yellow Leaf Curl Virus (TYLCV, GenBank Accession NC004005). All protein coding genes of this virus have been experimentally determined (Gronenborn 2007), with the presence of an overprinted gene and genes with large overlaps (Fig. 1A).

Unsurprisingly, Prodigal and PHANOTATE do not correctly predict all of these six known genes (Fig. 1B and C). A set of 1106 sequences similar to TYLCV were collected from the nr database (BLASTn, 90 ID%) and mapped to the TYLCV genome, allowing the detection of 3886 SNPs (Fig. 1E and F). From these SNPs, a pNeg/pS ratio was computed for the 28 TYLCV predicted ORFs (Fig. 1D). The best candidate coding regions are the ten ORFs with a pNeg/pS ratio below or equal to 1, in addition to one with a 1.09 ratio. Yet, as synonymous mutations often involve the third nucleotide of codons (128 out of the 193 times), the presence of a coding region artificially leads to mirrored synonymous mutations on one of the frames on the complementary strand. On the 11 ORFs with a small pNeg/pS ratio, six are coded inside three larger ones on the opposite strand. As bidirectional coding is a rare phenomenon (Carter 2021), these six ORFs are automatically outlined in red and discarded (Fig. 1D). The five remaining ORFs correspond to five of the six known genes of TYLCV. The remaining gene is C4, nested within the replication protein on an alternate reading frame, and is the only region with ratio lower to 1.65 (ratio of 1.35). Selection pressures on the ancestral gene constrain the nature of substitutions of an overprinted gene and lead to pNeg/pS ratios greater than 1 for overlapping genes (Pavesi 2006, Olo Ndela *et al.* 2023). Thus, ORFs with values up to 1.5 should be considered for overprinted regions. Additionally, the ORF corresponding to the V2 gene has its start position 42 nt upstream the true start position. To detect these issues, CodingDiv computes the pNeg/pS for the first 50 nucleotides of each ORF and if this value is at least twice greater than the one of the whole ORF, the start position of the ORF is reported as potentially erroneous. For the ORF corresponding to V2, the pNeg/pS value is 3.7 in the noncoding stretch at the beginning of the ORF and 0.57 in the whole ORF, a red square was thus added at the beginning of the ORF (Fig. 1D). To illustrate CodingDiv results on an uncultivated phage, the single cell amplified genome vSAG 37-F6 (GenBank accession KY052810), abundant in marine metagenomes (Martinez-Hernandez *et al.* 2017), was analyzed and described in the Supplementary Material.

## 4 Conclusion

CodingDiv allows to refine gene prediction by using (meta)genomic microdiversity to separate coding from noncoding regions, even for small genes and overlapping ones. As it uses gene selection pressures, CodingDiv can be applied to genomes of all nature (RNA, DNA, linear or circular) as long as it encodes proteins. We believe that CodingDiv will help decipher the complex structure of viral genomes.

## Supplementary Material

btad408_Supplementary_Data

## Data Availability

The data underlying this article are available in the article and in its online supplementary material.
