# Supplementary material for: CodingDiv: analyzing SNP-level microdiversity to discriminate between coding and noncoding regions in viral genomes
Source: Bioinformatics. 2023 Jul 14;39(7):btad408. doi: 10.1093/bioinformatics/btad408 (PMC10356776; doi:10.1093/bioinformatics/btad408)
Supplement: btad408_Supplementary_Data [file btad408_supplementary_data.pdf]

Supplementary material

In addition to being able to analyze cultured viruses for which similar genomes can be found in GenBank, such as the TYLCV virus described in the main text, metagenomic contigs can also be analyzed with CodingDiv. Here, we present the analysis of vSAG37_F6, an assembled contig, for which SNPs were identified by using metagenomic reads. Both examples account for the two usage scenarios of CodingDiv.

The genome of vSAG 37-F6 (13,589 bp) was predicted to encode 23 and 27 proteins by Prodigal and PHANOTATE respectively, all of them being on the reverse strand. The 23 proteins predicted by Prodigal were also predicted by PHANOTATE with the same coordinates, except for two proteins that were shorter in PHANOTATE (28 and 32 amino acids shorter respectively). The four additional proteins predicted by PHANOTATE are small proteins (<43 amino acids, or AA) encoded in intergenic regions of the 23 genes. This result is expected, as the PHANOTATE algorithm is meant to maximize the coding potential of a given nucleotide sequence.

Considering ORFs, a total of 98 were predicted (>100 nt), 51 on the forward strand and 47 on the reverse strand. Only relatively small ORFs were predicted on the forward strand (51 AA-long on average, only three longer than 85 AA) already hinting that coding regions are on the reverse strand (113 AA-long on average, 20 longer than 85 AA). In addition, the 22 ORFs with the smallest pNeg/pS values (between 0.03 and 0.24) are all encoded on the reverse strand.

Considering the reverse strand only, 28 out of 47 ORFs have pNeg/pS values lower than 1. Out of these 28 ORFs supposed to be under purifying selection, (i) 23 correspond to Prodigal predictions, (ii) three to genes predicted only by PHANOTATE and (iii) two ORFs not predicted by Prodigal or PHANOTATE. Among these two last ORFs, one is likely a coding ORF as it is encoded in an intergenic region with an overlap of only one nucleotide with another ORF and is 50 AA-long. The last ORF not predicted by any software and with a good pNeg/pS value is a 37 AA-long ORF overprinted inside a 333 AA-long one.

On the forward strand, 24 of the 51 ORFs have pNeg/pS ratios below 1 (depicted in blue in Fig. Supp. 1). As previously described for TYLCV, the small pNeg/pS values of these 24 ORFs are likely due to mirrored mutations, the presence of synonymous mutations on a coding ORF leading to an over-representation of synonymous mutations on the paired frame on the opposite strand. Paired frames, that depend on the genome length, were thus identified and indicated by three colors (pale blue, red and yellow) on the boxes at the right of each substitutions bar chart. Considering the 24 ORFs with low pNeg/pS ratios on the forward strand, 21 ORFs indeed correspond to the paired frame of larger coding regions with small pNeg/pS ratios on the reverse strand. These 21 ORFs are thus depicted with red edges (Fig. Supp. 1). It has to be noted that the ORFs that should be depicted with red edges are not always correctly predicted, as a coding ORF can be shorter than a non-coding ORF situated on its paired frame. Thus, three blue-colored ORFs on the positive strand are incorrectly depicted with black edges and three blue-colored ORFs on the reverse strand have red edges. Even though this edge coloring is not perfect, we believe it is important as it points out this issue to the user.

As a conclusion, ORF length and pNeg/pS points towards the fact that protein coding

genes are only encoded on the reverse strand. All the 23 protein coding genes predicted by Prodigal have small pNeg/pS values and can be considered as *bona fide* genes. Yet, as many as five additional ORFs have good mutation signals and can be considered as likely coding. As expected, Prodigal is specific but not sensitive for small genes. Unsurprisingly, PHANOTATE predicted more genes (27) and 26 seem as likely *bona fide* genes. Yet, one of these genes has a very high pNeg/pS ratio (2.8, with 25 SNPs leading to mutations with negative scores, including 5 mutations of an AA to a stop codon, and only 9 synonymous mutations) and is likely a false positive. Thus, in this example, PHANOTATE is unsurprisingly more sensitive than prodigal, but at the expense of the specificity. In addition, two ORFs not predicted by the gene prediction tools have low pNeg/pS ratio and are likely corresponding to existing genes.

In addition to helping identify all the genes encoded in this genome, the mutation signal can also help in the identification of the correct position of their start codons. As previously seen, the 23 genes predicted by Prodigal and PHANOTATE have the same start codon positions, except one. For this gene encoded on the -3 frame, the predicted start codon positions are 7818 and 7722 by Prodigal and PHANOTATE respectively, and 7887 for the corresponding ORF. Coherently with software predictions, this start position for this ORF is reported by CodingDiv as potentially erroneous as the pNeg/pS value for its first 50 nucleotides is more than twice greater than the value for the whole ORF. Indeed, 17 non synonymous "negative" SNPs were detected on the first 50 nucleotides (pNeg/pS of 8.5) and only 2 synonymous ones, compared to 33 and 46 on the last 240 nucleotides (pNeg/pS of 0.71, pNeg/pS of 1.04 for the whole ORF).
To further try and detect the correct start position of the coding region corresponding to the ORF73, a representation of the SNPs and of their effect on the amino acids potentially encoded was generated (Figure S2). In Region 1 (position 7887-7852 of the genome), the mutations are mostly synonymous ones for ORF72 and non synonymous ones with negative scores for ORF73. This genome region is clearly only coding on the -2 frame and the beginning of the ORF73 (position 7887) is not the correct position of the start codon. Concerning Region 3 (position 7793-7693), it is only potentially coding on the -3 frame, and it indeed seems to be coding as the mutations for the ORF73 in this region are mostly synonymous. The start codon predicted by PHANOTATE situated in this zone (position 7722) is not the correct one, and this start codon is thus likely in Region 2.
In this region 2 where two genes are overlapping, selection pressures seem here to be mostly applied to the -2 frame, with an overabundance of synonymous mutations. This likely represents the ancestral coding region. On the -3 frame on this Region 2, mutations are mostly negative ones but with an absence of mutations with strongly negative scores and mutations leading to the apparition of a Stop codon, as it is the case in the non coding Region 1 for the -3 frame in which SNPs lead to two very negative mutations and three stop codons. Considering that Region 2 is already coding on the -2 frame, this pattern is not surprising for an overlapping part on the -3 frame, the beginning of the ORF73 being likely more flexible structurally. Interestingly, one SNP that impacts the amino acid between Region 1 and 2 leads to a stop codon, and this SNP is found on 407 out of 1478 reads mapped to genome position. This tends to indicate that almost a third of the viruses

of this population have a shorter version of the ORF72 and could be a way to modulate its gene content at the population level.

Because viruses present distinct mutation rates depending on their nucleic acid, CodingDiv was also tested on the RNA genome of the Human T-Lymphotropic Virus 1 (HTLV1) known to exhibit high mutation rates (Duffy 2018). Notably, protein coding regions of HTLV1 present an enrichment in synonymous mutations and a depletion in negative substitutions (Fig. S3). Thus, the substitution signal seems to be similar between RNA viruses and single- or double-stranded DNA viruses despite variable mutation rates, making CodingDiv suitable for all types of viral nucleic acid. Prodigal and PHANOTATE predict 8 and 12 total genes respectively, but, of course, fail to correctly predict spliced genes. Because of its overprinted nature, both tools completely missed the *rex* gene, whereas the longer 3′ end of the tax gene was predicted. The spliced 5′ parts of genes *rex*, *tax* and *hbz* were also missed, again, likely due to overprinting of the 5′ end of *ref/tax* and the very small size of the 5′ end of *hbz* (11 additional nucleotides). In addition, genes were overpredicted in the long terminal repeats (LTR) although these regions are non-coding by definition. CodingDiv on the other hand, when lowering the ORF minimum size to 30 nucleotides, manages to find the 5′ additional sequence of *rex* and *tax*, with a pNeg/pS ratio of 1, while the beginning of the *hbz* gene is predicted but deprived of SNPs. The 3′ longer spliced part of the *rex* gene has an aberrant pNeg/pS value of 7.5, comparable to ratios observed for non-coding ORFs, way past the announced maximum of 1.5 for overprinted genes. This outlier is due to the particularly relaxed selective constraints applied to the *rex* gene thus capable of withstanding more amino acid changes, as described in (McGirr and Buehuring 2006). Nonetheless, 8 ORFs predicted on the long terminal repeats (LTR) present pNeg/pS values below 1. This observation is not surprising as (i) LTRs are known to be evolutionary conserved (Benachenhou et al. 2009) and (ii) genes *tax* and *hbz* overlap the 3′ LTR region, resulting in a functional constraint applied to this region. Outside of LTRs, other false positive ORFs are predicted with low pNeg/pS (between 1 and 0.6) on the negative strand. The small size of the latter could be enough to discard them, but very short ORFs were predicted to not miss spliced regions thus increasing the risk of false positives as one or two hazardous synonymous SNPs in a small region can randomly result in good pNeg/pS values.

<u>Additional references</u>:

Benachenhou F, Jern P, Oja M, *et al.* Evolutionary Conservation of Orthoretroviral Long Terminal Repeats (LTRs) and ab initio Detection of Single LTRs in Genomic Data. *PLoS ONE* 2009;**4(4)**:e5179.

Duffy S. Why are RNA virus mutation rates so damn high?. *PLOS Biology* 2018;**16(8)**: e3000003

McGirr KM, Buehuring GC. Tax & Rex: Overlapping Genes of the Deltaretrovirus Group. *Virus Genes 2006;***32**:229–239.

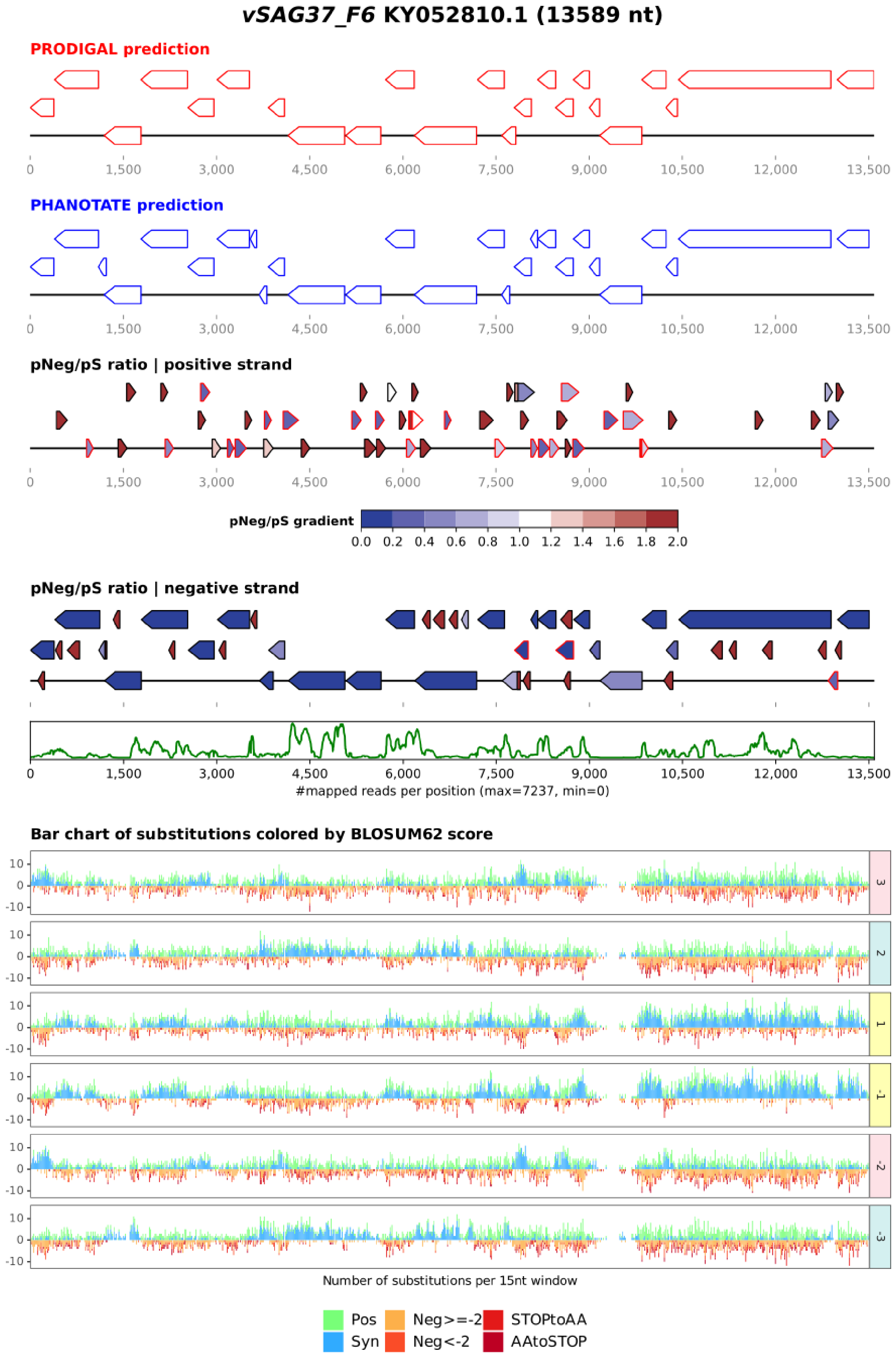
vSAG37_F6 KY052810.1 (13589 nt)
PRODIGAL prediction
PHANOTATE prediction
pNeg/pS ratio | positive strand
pNeg/pS gradient
pNeg/pS ratio | negative strand
#mapped reads per position (max=7237, min=0)
Bar chart of substitutions colored by BLOSUM62 score
Number of substitutions per 15nt window
Pos
Syn
Neg>=-2
Neg<-2
STOPtoAA
AAtoSTOP

Figure S1: Image, in SVG format, generated by CodingDiv when ran on vSAG 37-F6 virus (#KY052810). Virome reads from a surface water sample collected in the South Atlantic Ocean (the Tara Oceans expedition, position 076, Gregory et al. 2019) were mapped to detect SNPs (2 reads minimum for a SNP to be called, representing 1% of sequencing depth) and assess microdiversity. The genomic maps and plots described in Figure 1 can be found, i.e. protein prediction with Prodigal and PHANOTATE, ORF prediction (100 nt at least) colored according to their pNeg/pS ratios and mapping coverage and bar chart counting substitutions from each SNP category.

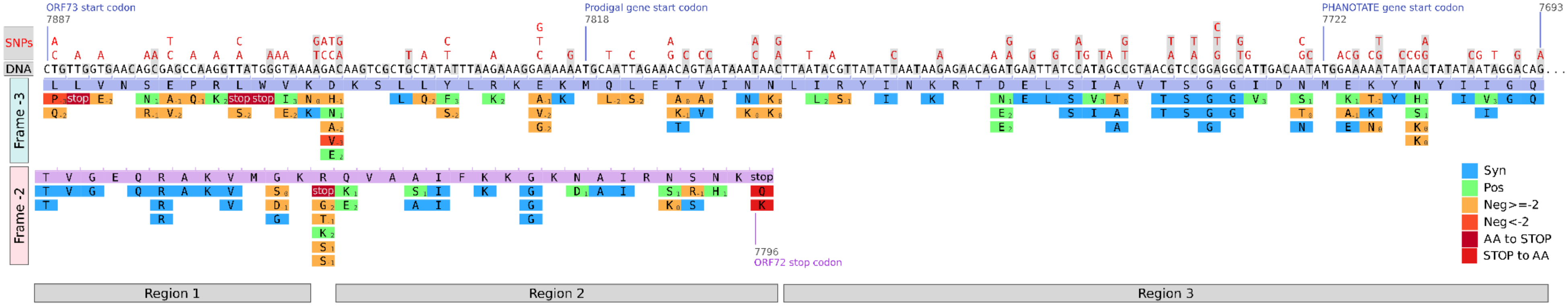

Figure S2: Graphical representation of the portion of the vSAG 37-F6 genome between the position 7693 and 7887, and of the SNPs detected in mapped virome reads (tracks “DNA” and “SNPs” at the top). The negative strand of this genome region is here shown, but positions are referring to the positive strand and are thus decreasing. The ORF73 and ORF72, encoded respectively on frame -3 and -2 are shown in pale blue and red respectively. The mutation of each SNP on the amino acid level of these two ORFs are indicated right below these ORFs and colored according to their type (synonymous mutations in blue, non synonymous mutation with a positive BLOSUM62 score in green, with negative scores in orange and red and mutations transforming a residue into a stop codon or a stop codon into a residue in dark red). Position of the start codon and eventually of the stop codon of the two ORF are indicated, as well as the position of genes predicted by Prodigal and PHANOTATE.

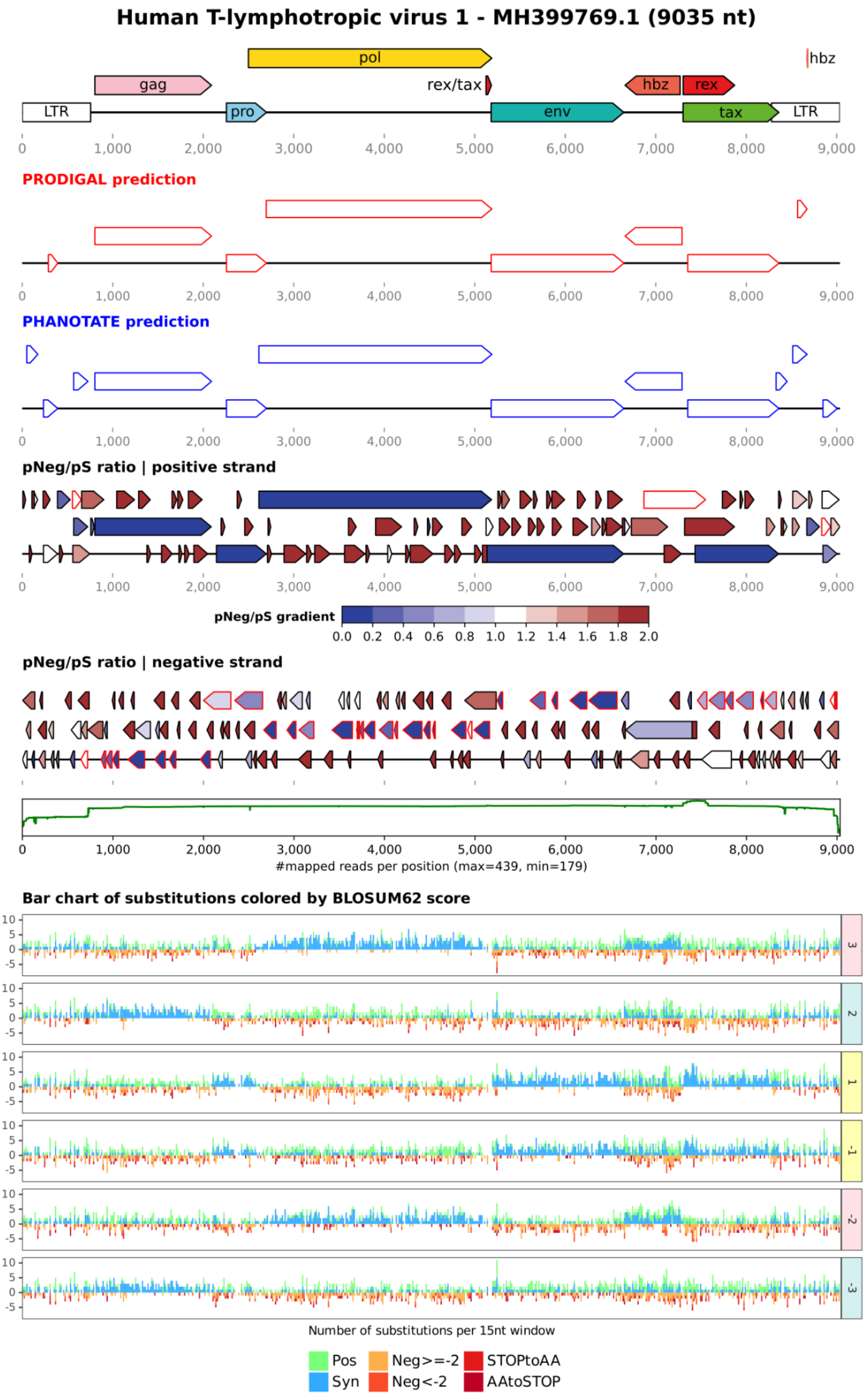

Human T-lymphotropic virus 1 - MH399769.1 (9035 nt)
pol
gag
rex/tax
hbz
hbz
rex
LTR
pro
env
tax
LTR
0
1,000
2,000
3,000
4,000
5,000
6,000
7,000
8,000
9,000
PRODIGAL prediction
PHANOTATE prediction
pNeg/pS ratio | positive strand
pNeg/pS gradient
0.0 0.2 0.4 0.6 0.8 1.0 1.2 1.4 1.6 1.8 2.0
pNeg/pS ratio | negative strand
#mapped reads per position (max=439, min=179)
Bar chart of substitutions colored by BLOSUM62 score
10
5
0
-5
3
2
1
-1
-2
-3
Number of substitutions per 15nt window
Pos
Syn
Neg>=-2
Neg<-2
STOPtoAA
AAtoSTOP

Figure S3: Image, in SVG format, generated by CodingDiv when ran on Human T-Lymphotropic Virus 1 (#MH399769). 609 complete genomes of deltaretroviruses were retrieved from GenBank and mapped to the reference genome to detect SNPs (2 reads minimum, representing 1% of sequencing depth). A minimum ORF size of 30 nucleotides was set to have access to smaller spliced ORFs. Genomic maps and plots described in Figure 1 can be found here, *i.e.* protein prediction with Prodigal and PHANOTATE, ORF prediction (100 nt at least) colored according to their pNeg/pS ratios and mapping coverage and bar chart counting substitutions from each SNP category. Note: ORFs coordinates are not all coherent with the reference genomic map because of splicing. *Getorf* looks for start codons while spliced parts do not necessarily begin with a Methionine.
